# Differential impact of pegfilgrastim, a recombinant human granulocyte colony stimulating factor, on the neutrophil count of male and female deer mice *(Peromyscus maniculatus bairdii)*

**DOI:** 10.1186/s40360-024-00778-y

**Published:** 2024-08-19

**Authors:** J.P. Strydom, Linda Brand, Francois P. Viljoen, De Wet Wolmarans

**Affiliations:** Center of Excellence for Pharmaceutical Sciences, Department of Pharmacology, Faculty of Health Sciences, North-West University, Building G23, Office 315, 11 Hoffman Street, Potchefstroom, 2531 South Africa

**Keywords:** Neutrophil-lymphocyte ratio, Deer mouse, Pegfilgrastim, G-CSF, Compulsive, Animal model

## Abstract

**Background:**

An increasing body of research implicates inflammatory processes, including alterations in the neutrophil-lymphocyte ratio (NLR), in the pathophysiology of psychiatric illness. The deer mouse *(Peromyscus maniculatus bairdii)* is commonly studied for its naturalistic expression of compulsive-like behaviour. Towards future efforts to gain an understanding of how innate and adaptive immune processes might be involved in this model, we aimed to study the effects of pegfilgrastim, a pegylated recombinant human granulocyte colony-stimulating factor (g-CSF) analogue, on the NLR of both male and female deer mice.

**Methods:**

Briefly, 54 deer mice (equally distributed between sexes) were exposed to a single injection with either control or pegfilgrastim (0.1 or 1 mg/kg) (*n* = 18 per group). Six mice of each group (three per sex) were euthanized on days two, four and seven post-administration, their blood collected and the NLR calculated. Data were analysed by means of ordinary three-way ANOVA, followed by Bonferroni post-hoc testing.

**Results:**

Irrespective of dose, pegfilgrastim resulted in higher NLR values in mice of both sexes at days four and seven of testing. However, female mice exposed to the higher dose, presented with significantly higher NLR values irrespective of time, compared to male mice exposed to the same.

**Conclusion:**

The data generated from this work highlight important dose- and sex-specific aspects of pegfilgrastim with female mice showing heighted elevation of the NLR in response to high-dose pegfilgrastim administration only. Since the innate immune components of male and female deer mice is differentially sensitive to g-CSF stimulation, our results provide a useful basis for further study of sex-specific immunological processes in deer mice.

## Background

An increasing body of research implicates inflammatory processes in the aetiology and pathophysiology of psychiatric illness [1]. With respect to obsessive-compulsive disorder (OCD), clinical data implicate immune system involvement in specific cohorts. For example, children diagnosed with paediatric autoimmune neuropsychiatric disorders associated with streptococcal infection (PANDAS) and paediatric acute-onset neuropsychiatric syndrome (PANS) present with sudden-onset compulsive symptoms following said immunogenic hit [2]. Also, elevated plasma inflammatory cytokine concentrations were observed in adult cohorts in the absence of prior infectious history or other clinical comorbidities [3]. In contrast, reductions in inflammatory markers were also noted [4], while a recent systematic review and meta-analysis of the available literature failed to confirm immune dysregulation in OCD [5]. While such disparity in findings can likely be ascribed to differences in research methodologies followed in the various investigations [5], a clear understanding of how immunological mechanisms may underlie compulsive symptom expression is further clouded by therapeutic findings. Indeed, the clinical efficacy of immunomodulation, though currently recommended for the treatment of PANDAS/PANS [6], remains suboptimal [7].

One proposed marker of heightened inflammatory responses is an increased neutrophil-lymphocyte ratio (NLR) [8] which rather than being illness-specific, is a broad indication of mostly innate immune activation that occurs in both infective [9] and non-infective [10] conditions. Although widely investigated in conditions not related to the central nervous system [11], increased NLR values have also been observed in neuropsychiatric cohorts, e.g. patients suffering from schizophrenia, bipolar disorder, major depressive disorder [8], and OCD [12].

While increased NLR values do not associate with specific conditions *per se*, manipulations of the NLR in animal model systems might be informative for an understanding of how innate and acquired immune components may interact to modulate the expression of specific behavioural phenotypes. To this end, naturalistic animal models that mimic specific biobehavioural aspects of human neuropsychiatric conditions, could be useful [13]. One example is the deer mouse *(Peromyscus maniculatus bairdii)*, a wildtype model system that has been extensively validated in terms of its resemblance of compulsive-like behavioural expression. When bred, reared, and housed under standard laboratory conditions, approximately 40% of deer mice of both sexes spontaneously develop high stereotypical behaviour (HS). HS behaviour is repetitive and persistent, occurs in the absence of a clear goal, and waxes and wanes throughout the course of a wake cycle [14] (for a detailed review, see Scheepers, Stein [15]). Two main stereotypical phenotypes have been described, i.e. vertical jumping and pattern running stereotypies [16]. Said behaviours also resemble OCD in terms of its neurobiological underpinnings and response to pharmacotherapy. Specifically, HS is founded upon similar cortico-striatal perturbations than clinical OCD [14, 17] and is modestly responsive to chronic, high-dose exposure to the selective serotonin reuptake inhibitor, escitalopram [16]. While changes in immune system activation and white cell proliferation in deer mice have been studied against the background of environmental fluctuation before (see Demas and Nelson [18] and others), no investigation has yet explored the potential effects of pharmacological NLR manipulation on the behavioural output in this species.

Since we apply deer mice of both sexes in our laboratory to model specific aspects of compulsive-like behavioural persistence, and given that we, as part of ongoing work, intend to investigate the potential effects of NLR manipulation on stereotypical motor expression, we first aimed to explore the effects of pegfilgrastim, a pegylated recombinant human granulocyte colony-stimulating factor (g-CSF) analogue [19], on the NLR of both male and female deer mice. This approach was necessary for two reasons. Firstly, most prior investigations relating to the development [20] of pegfilgrastim and its application in other mouse strains [21], were completed in male mice. Secondly, longitudinal, neurodevelopmental investigations into the effects of NLR manipulation on adult deer mouse behaviour will be contingent on the chronic manipulation of immune functioning. The present investigation thus employed pegfilgrastim, as opposed to its unpegylated form, filgrastim, since it showed a longer-lasting effect on the neutrophil count of male mice after a single injection in previous work [20]. This reduces the need for multiple injections administered over a single week, thereby enabling long-term study.

## Methods

### Study layout

**Fig. 1:**
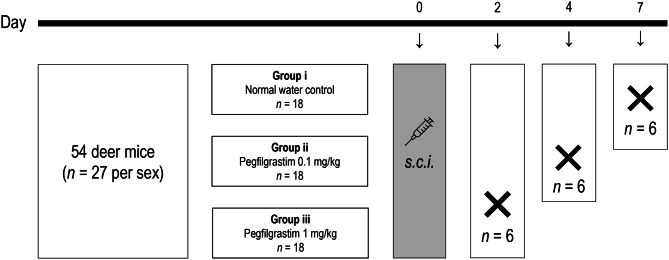
Schematic representation of experimental layout. s.c.i.: subcutaneous injection; crosses indicate euthanisation of six animals per group on days 2, 4, and 7, respectively

This investigation was conducted over seven days in fifty-four (54) deer mice aged 10–16 weeks (approximately 18 g) at the onset of investigation (Fig. 1). Mice were randomly divided into three different exposure groups (*n* = 18, per group, equally distributed between sexes), i.e. (i) normal water control, (ii) pegfilgrastim 0.1 mg/kg [20], and (iii) pegfilgrastim 1 mg/kg [20]. After a single injection, mice of each exposure group were euthanized in groups of *n* = 6 (*n* = 3 per sex) per exposure group on post-injection days two, four and seven, respectively.

### Animals and housing

Deer mice *(Peromyscus maniculatus bairdii)*, of which the progenitors were acquired from the *Peromyscus* Genetic Stock Center (University of South Carolina, USA), were bred, reared, and housed at the vivarium (SAVC reg no: FR15/13458; AAALAC accreditation file: 1717) of the North-West University (NWU), Potchefstroom, South Africa. Two days prior to experimental group allocation on experimental day 0, mice were randomised considering litter and rearing cage and reintroduced in groups of three same-sex animals per cage (see ‘Drug’ also). Cages [35 cm (l) × 20 cm (w) × 13 cm (h); Techniplast^®^ S.P.A., Varese, Italy] were individually ventilated, kept at 23 °C and a relative humidity of 60%, and maintained on a normal 12-h light/dark cycle (06:00/18:00). Food (standard rodent chow) and water were provided *ad lib*. Cages were equipped with paper towel as a form of nesting material and a 10 cm-long piece of polyvinylchloride pipe as a form of environmental enrichment. All experiments were approved by the AnimCare research ethics committee of the North-West University (approval number: **NWU-00760-22-A5**) and were carried out in accordance with the South African National Standard 10386: ‘The Care and Use of Animals for Scientific Purposes’.

### Drug exposure

Pegfilgrastim (Pegylastin^®^, 0.6 mL of a 10 mg/mL ready-to-inject solution) was acquired from CJ Pharmaceutical Enterprises LTD (Delmas, South Africa). To constitute smaller stock solutions, 100 µL of the marketed formula were diluted to a volume of 5 mL with water for injection (Fresenius-Kabi^®^, Qheberha, South Africa). The resulting solution was slowly stirred by vortex and used to administer the dose of 1 mg/kg dose. Another identical stock solution was further diluted to 50 mL using water for injection; this was used to administer the 0.1 mg/kg dose. These doses were based on previous pre-clinical study in mice [20]. Since the individual weights of mice varied between 14 g and 22 g, the injection volume was never more than 110 µL, administered into the interscapular subcutaneous tissue. All stock solutions were freshly prepared on experimental day 0. Mice injected with control received water for injection at the same volume-per-weight ratio as used for pegfilgrastim. To prevent housing bias from confounding the results, each mouse in a specific housing cage was ear-tagged for identification purposes and assigned to a different exposure group.

### Sample collection and slide preparation

On experimental days two, four and seven, the required number of animals were euthanised by means of cervical dislocation without prior exposure to an anaesthetic [22] and decapitated by means of a rapid guillotine cut. After decapitation, trunk blood was collected in ethylenediaminetetraacetic acid-containing microtainers. Immediately following the collection of trunk blood, a single drop of whole blood was transferred onto a glass slide, using a capillary pipette. Smears were subsequently prepared according to the method of Houwen [23].

### Leukocyte staining and differential cell counting

Leukocyte staining was performed using a standard Hemacolor^®^ staining kit (Merck^®^, Johannesburg, South Africa) as specified by the manufacturer. Neutrophil and lymphocyte counts were subsequently conducted using a trinocular light microscope (B-190 series, 60 × magnification, OPTIKA^®^ Italy, Ponteranica, Italy), following the prescribed protocol of Koepke [24]. Differential counts of 100 cells on each slide were performed twice by a blind observer. If the two NLR values varied by more than 25%, slides were recounted for a third time. Counting results were recorded as percentages which were used to calculate the NLR (final counted values shown in Table 1).

**Table 1:** Neutrophil and lymphocyte counts in male and female mice exposed to a single control or pegfilgrastim (0.1 or 1 mg/kg) subcutaneous injection at day two, four and seven post-administration

Day	Dose (mg/kg)	Sex	Neutrophils	Lymphocytes	NLR
2	0	M	24	75	0.320
		M	32	61	0.525
		M	32	64	0.500
		F	21	76	0.276
		F	14	82	0.171
		F	26	67	0.388
	0.1	M	33	61	0.541
		M	32	65	0.492
		M	23	71	0.324
		F	28	67	0.418
		F	26	68	0.382
		F	34	61	0.557
	1	M	28	68	0.412
		M	30	66	0.455
		M	29	64	0.453
		F	33	57	0.579
		F	32	63	0.508
		F	35	57	0.614
4	0	M	22	74	0.297
		M	13	84	0.155
		M	24	73	0.329
		F	15	82	0.183
		F	24	72	0.333
		F	17	78	0.218
	0.1	M	47	50	0.940
		M	32	65	0.492
		M	36	60	0.600
		F	38	59	0.644
		F	39	59	0.661
		F	34	61	0.557
	1	M	34	63	0.540
		M	22	72	0.306
		M	47	50	0.940
		F	53	44	1.205
		F	51	44	1.159
		F	50	47	1.064
7	0	M	13	86	0.151
		M	22	71	0.310
		M	17	78	0.218
		F	24	69	0.348
		F	15	83	0.181
		F	13	82	0.159
	0.1	M	36	60	0.600
		M	52	45	1.156
		M	30	64	0.469
		F	30	67	0.448
		F	24	75	0.320
		F	31	65	0.477
	1	M	13	86	0.151
		M	44	55	0.800
		M	44	54	0.815
		F	41	55	0.745
		F	46	48	0.958
		F	51	45	1.133

*M* male, *F* female, *NLR* neutrophil-lymphocyte ratio. Data representative of cell counts of up to 100 cells for each sample

### Statistical analysis

All statistical analyses were carried out with IBM^®^ SPSS^®^ version 28 (IBM^®^ Software, New York, US), whereas graphs were prepared with Graphpad^®^ Prism^®^ version 9 (Graphpad^®^ Software, San Diego, US). The sample size in this study was estimated based on (i) the presumption that a singular pharmacological intervention with a known mechanism of action was used in wildtype mice that had the same prior experience, as was shown in our laboratory before [25] and (ii) the fact that a single dependent variable, i.e. NLR was measured. Thus, a similar biological response to each dose of said intervention could reasonably be predicted. To determine the potential interactions between exposure, sex, and time, ordinary three-way analysis of variance (ANOVA) was run, followed by interpretations of two-way interactions and main effects, where applicable. Bonferroni post-hoc comparisons were used to compare group means. Statistical significance was set at *p* < 0.05 all analyses. Cohen’s *d* effect size calculations were applied to highlight the magnitude of group differences observed [26].

## Results

The mean NLRs quantified over the three respective timepoints for mice in the control group were consistent with prior reports [27] (D2: 0.36 ± 0.13; D4: 0.25 ± 0.08; D7: 0.23 ± 0.08).

**Fig. 2:**
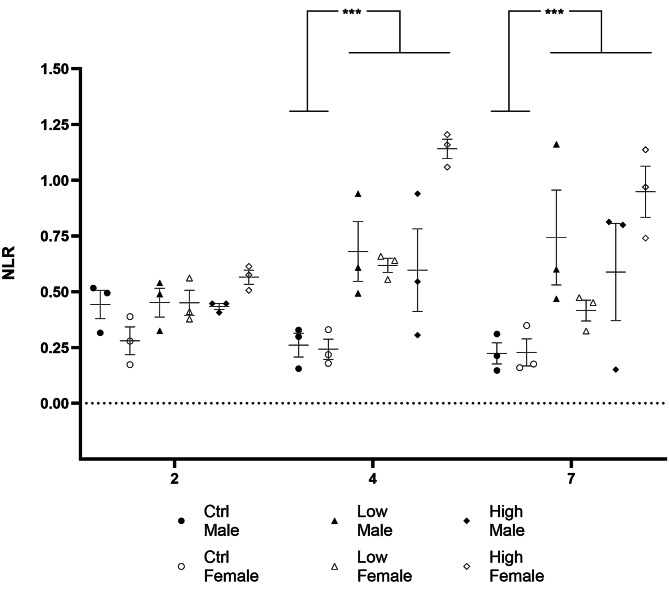
NLR values of male and female deer mice exposed to a single s.c.i with either control or pegfilgrastim (0.1 and 1 mg/kg) as calculated at two, four, and seven days post injection. Data are reflective of individual mice, with lines indicating mean ± standard deviation. Only the main effects of dose at day four and seven are shown. All other descriptive statistics are provided in Table 2. Three-way ANOVA followed by Bonferroni. *p* ≤ 0.004. *NLR* neutrophil-lymphocyte ratio

Exposure, sex and time did not show a significant three-way interaction in terms of impacting the NLR (*F*[4, 36] = 1.35, *p* = 0.27) (Fig. 2). However, exposure interacted significantly with sex (*F*[2, 36] = 9.32, *p* < 0.001) and time (*F*[4, 36] = 3.23, *p* = 0.02), respectively. Specifically, irrespective of time, the NLR values of male and female mice differed significantly at the level of high dose pegfilgrastim only (*p* < 0.001), with this difference missing statistical significance with respect to the low dose (*p* = 0.13). However, the NLR values of both male and female mice exposed to the low and the high dose of pegfilgrastim differed significantly from their respective sex-matched controls (**0.1 mg/kg**: male: *p* = 0.002, female: *p* = 0.02; **1 mg/kg**: male: *p* = 0.03, female: *p* < 0.001). In terms of the exposure-time interaction, no differences were observed between any of the exposure groups on day two. However, both doses resulted in increased NLR values on day four (**0.1 mg/kg**: *p* = 0.001; **1 mg/kg**: *p* < 0.001) and day seven (**0.1 mg/kg**: *p* = 0.004; **1 mg/kg**: *p* < 0.001), compared to the control. Also, over time, only the high-dose pegfilgrastim increasingly bolstered the NLR through day seven, with differences seen between the NLR values of mice at both days four (*p* = 0.003) and seven (*p* = 0.04), compared to day two. The same comparisons were insignificant for the low-dose-exposed group. For a complete overview of the descriptive statistics, significance, and effect sizes of pairwise comparisons, please refer to Table 2.

**Table 2:** Descriptive statistics of pairwise comparisons (significant *p*-values or large Cohen’s d effect size values are highlighted in bold)

	Comparison	NLR (Mean ± SD)	*p*	*d*	95CI
**Dose × Day**	***Day 2 vs 4***				
	Ctrl	0.36 ± 0.13 vs 0.25 ± 0.08	0.880	**1.0**	−2.205—0.222
	Low	0.45 ± 0.09 vs 0.65 ± 0.15	0.185	**1.6**	0.206—2.836
	High	0.50 ± 0.08 vs 0.87 ± 0.36	**0.003**	**1.4**	0.092—2.661
	***Day 2 vs 7***				
	Ctrl	0.36 ± 0.13 vs 0.22 ± 0.08	0.587	**1.2**	−2.448—0.050
	Low	0.45 ± 0.09 vs 0.58 ± 0.30	0.654	0.6	−0.590—1.729
	High	0.50 ± 0.08 vs 0.77 ± 0.33	**0.039**	**1.1**	−0.147—2.310
	***Day 4 vs 7***				
	Ctrl	0.25 ±.077 vs 0.22 ± 0.08	1.000	0.3	−1.452—0.828
	Low	0.65 ± 0.15 vs 0.58 ± 0.30	1.000	0.5	−1.632—0.671
	High	0.87 ± 0.36 vs 0.77 ± 0.33	1.000	0.3	−1.419—0.858
	***Ctrl vs Low***				
	Day 2	0.36 ± 0.13 vs 0.45 ± 0.09	1.000	**0.8**	−0.419—1.943
	Day 4	0.25 ± 0.08 vs 0.65 ± 0.15	**0.001**	**3.3**	1.418—5.025
	Day 7	0.22 ± 0.08 vs 0.58 ± 0.30	**0.004**	**1.6**	0.258—2.918
	***Ctrl vs High***				
	Day 2	0.36 ± 0.13 vs 0.50 ± 0.08	0.563	**1.3**	−0.020—2.492
	Day 4	0.25 ± 0.08 vs 0.87 ± 0.36	**< 0.001**	**2.4**	0.798—3.838
	Day 7	0.22 ± 0.08 vs 0.77 ± 0.33	**< 0.001**	**2.2**	0.706—3.675
	***Low vs High***				
	Day 2	0.45 ± 0.09 vs 0.50 ± 0.08	1.000	0.6	−0.617—1.696
	Day 4	0.65 ± 0.15 vs 0.87 ± 0.36	0.119	**0.8**	−0.413—1.951
	Day 7	0.58 ± 0.30 vs 0.77 ± 0.33	0.225	0.6	−0.579—1.742
**Dose × Sex**	***Female vs Male***				
	Ctrl	0.25 ± 0.08 vs 0.31 ± 0.13	0.489	0.5	−0.423—1.460
	Low	0.49 ± 0.12 vs 0.62 ± 0.26	0.130	0.6	−0.316—1.584
	High	0.88 ± 0.28 vs 0.54 ± 0.26	**< 0.001**	**1.3**	−2.294— −0.245
	***Ctrl vs Low***				
	Female	0.25 ± 0.09 vs 0.49 ± 0.11	0.018	**2.4**	1.118—3.582
	Male	0.31 ± 0.13 vs 0.62 ± 0.26	0.002	**1.5**	0.449—2.575
	***Ctrl vs High***				
	Female	0.25 ± 0.09 vs 0.88 ± 0.28	**< 0.001**	**3.1**	1.665—4.480
	Male	0.31 ± 0.13 vs 0.54 ± 0.26	**0.028**	**1.1**	0.103—2.105
	***Low vs High***				
	Female	0.50 ± 0.12 vs 0.88 ± 0.28	**< 0.001**	**1.8**	0.700—2.938
	Male	0.62 ± 0.26 vs 0.54 ± 0.26	0.948	0.3	−1.253—0.608

## Discussion

The main aim of this investigation was to investigate the effects of different doses of pegfilgrastim on the NLR of male and female deer mice. The main findings of the present work are (1) that both doses of pegfilgrastim, compared to the control, resulted in higher NLR values in mice of both sexes at days four and seven of testing, and (2) female mice exposed to 1 mg/kg pegfilgrastim, presented with significantly higher NLR values irrespective of time, compared to male mice exposed to the same.

In terms of our first main result, we show that both doses of pegfilgrastim increased the NLR in deer mice of both sexes at days four and seven of testing (Fig. 2, Table 2). While the effect of pegfilgrastim on granulocyte counts were expected [20], earlier work in male C57bl/6 mice and male Sprague Dawley rats showed a similar effect already 12-h after administering pegfilgrastim at doses between 0.05 and 1 mg/kg in the mice and rats respectively [20, 28]. Further, that a sustained effect of pegfilgrastim on the NLR was observed in the present work until day seven post-administration, also somewhat contrasts with previous findings showing the neutrophil count to return to baseline by this time post-administration of a 1 mg/kg dose and even earlier after a 0.1 mg/kg dose [20]. The reasons for these temporal differences observed in deer mice might be multifactorial. For example, the delayed increase in the NLR could be related to slower absorption of the drug from the intrascapular injection site in deer mice. While the present work did not include a pharmacokinetic profiling of pegfilgrastim, prior work by Scholz, Ackermann [29] might be informative. Briefly, in a comparative study of the effects of filgrastim and pegfilgrastim, the authors showed that the transition of pegfilgrastim from the injection site to the plasma may be slower compared to what is true for filgrastim. Also, although Molineux, Kinstler [20] administered pegfilgrastim via subcutaneous injection at the interscapular site, Tanaka, Satake-Ishikawa [28] did so intravenously, which could have accounted for the rapid onset of action observed at the time. We also considered that the delay in the onset of action reported in the present work could have resulted from a potential early, but transient increase in the lymphocyte count, since pegfilgrastim has been shown to elicit such a response in humans [30]. However, our data are not congruent with such a conclusion (Table 1). With respect to its sustained impact on NLR elevation, it should be noted that pegfilgrastim is mainly eliminated via neutrophil-mediated action [31], whereby increased absolute neutrophil counts are normally associated with an increased elimination rate. It is thus possible that, combined with a delayed peak concentration as referred to above, a higher dose of pegfilgrastim is eliminated slower compared to a lower dose, resulting in sustained stimulation of granulocyte release. Nevertheless, further study is needed to confirm this notion.

Our third main finding highlights a striking sex- and dose-specific effect of pegfilgrastim in deer mice. Specifically, female mice that were exposed to the high dose presented with significantly higher NLR values at days four and seven post-administration, compared to their male counterparts. Importantly, this result was observed in the absence of any female-male difference in NLR at baseline (Fig. 2, Table 2). Further, while the effect of the higher dose of pegfilgrastim was similar at both days four and seven in male and female mice, respectively (Fig. 2, Table 2), the effect of the lower dose in female mice was at its maximum on day four post-administration, returning to near-baseline levels at day seven. This data should be considered with care, especially since no single study has, as far as we are aware of, explored the effects of pegfilgrastim on animals of both sexes from the same species in a single experiment. With respect to this result in deer mice, our data are especially important, since it might point to specific interactions between the pharmacodynamic actions of the drug and the underlying biology of male and female deer mice. To clarify, sex has not been shown to influence the immunomodulatory effects of other, albeit climate-related, interventions in deer mice [18]. In other words, the effect of pegfilgrastim in this model system cannot merely be ascribed to the known, but sometimes inconsistent [32] pro- and anti-inflammatory roles of female and male sex hormones, respectively [32], since such effects were found to be negligible under the prior circumstances [18]. Again, the potential influence of pharmacokinetic factors on the overall effect of pegfilgrastim in deer mice alluded to earlier, might be different for female and male mice, whereby the absorption and clearance of the drug administered at a high dose may be slower in female, compared to male mice. Importantly, that treatment-naïve male and female deer mice presented with similar NLR throughout all days of testing, would lend support for such a pharmacokinetic, rather than another biological explanation. Also, in clinical work, subcutaneous pegfilgrastim administration was associated with a large degree of variability [20], but since gender seems to have no effect in healthy humans, these findings need further exploration to come to definitive conclusions.

Collectively, the present results may have significant implications for studies relating to brain-immune crosstalk in the deer mouse model of behavioural persistence. For example, it should be considered that male and female mice will respond differently to g-CSF exposure, potentially inducing unique immunological effects in mice of the respective sexes. Therefore, conclusions of causality will need careful consideration. Secondly, it is also possible that unique naturalistic interactions between brain function and sex-specific immune processes might underlie the baseline biobehavioural presentation of male and female deer mice. Indeed, it might be especially worth investigating if animals of both sexes that present with various degrees of persistent behavioural intensity [33], also show unique immune profiles as reflected by differences in the NLR. It suffices to say that for work aimed at a greater understanding of immunological mechanisms in the presentation of persistent behavioural phenotypes, the present data provide a valuable foundation for future study.

Our work is not without limitation. Firstly, the marked influence of sex on the effects of pegfilgrastim on the NLR of deer mice was unanticipated and thus, inclusion of a larger number of mice at each sampling point may have been fruitful to observe an overall drug effect at each time point. Secondly, antigen-driven inflammation was not the focus of the present work. In fact, the direct effects of elevated non-reactive neutrophil concentrations, as opposed to that of antigen-driven neutrophilia on brain-immune crosstalk in the deer mouse model remain to be established; said investigation would be vital, especially considering that immune-inflammatory profiles observed in specific subpopulations of OCD, are mostly related to antigen-triggered reactivity. Work towards this end is continuing.

## Conclusion

We show that deer mice respond in a dose-, time-, and sex-dependent manner to single dose pegfilgrastim administration. Specifically, our results show for the first time that the actions of pegfilgrastim might be delayed in deer mice and that female mice exposed to a higher dose of pegfilgrastim, present with a markedly pronounced increase in the NLR, compared to male mice. These data provide a useful basis for further study of sex-specific immunological and brain-immune crosstalk processes in deer mice.

## Data Availability

The datasets used and/or analysed during the current study are available from the corresponding author on reasonable request.

## Abbreviations

OCD: Obsessive-compulsive-disorder
PANDAS/PANS: Paediatric autoimmune neuropsychiatric disorders associated with streptococcal infection/paediatric acute-onset neuropsychiatric syndrome
NLR: Neutrophil-lymphocyte ratio
HS: High stereotypical
g-CSF: Granulocyte colony stimulating factor
ANOVA: Analysis of variance
